# Current Research and Potential Applications of the Concealed Information Test: An Overview

**DOI:** 10.3389/fpsyg.2012.00342

**Published:** 2012-09-12

**Authors:** Gershon Ben-Shakhar

**Affiliations:** ^1^The Hebrew University of JerusalemJerusalem, Israel

**Keywords:** the concealed information test, psychophysiological detection of deception, the guilty knowledge test, memory detection, the searching CIT

## Abstract

Research interest in psychophysiological detection of deception has significantly increased since the September 11 terror attack in the USA. In particular, the concealed information test (CIT), designed to detect memory traces that can connect suspects to a certain crime, has been extensively studied. In this paper I will briefly review several psychophysiological detection paradigms that have been studied, with a focus on the CIT. The theoretical background of the CIT, its strength and weaknesses, its potential applications as well as research finings related to its validity (based on a recent meta-analytic study), will be discussed. Several novel research directions, with a focus on factors that may affect CIT detection in realistic settings (e.g., memory for crime details; the effect of emotional stress during crime execution) will be described. Additionally, research focusing on mal-intentions and attempts to detect terror networks using information gathered from groups of suspects using both the standard CIT and the searching CIT will be reviewed. Finally, implications of current research to the actual application of the CIT will be discussed and several recommendations that can enhance the use of the CIT will be made.

Deception is a frequent, perhaps essential, feature of human behavior, which may be expressed in a variety of situations (e.g., Saxe, 1991). The frequent use of deception in social contexts highlights the importance of detecting deception. However, research on perceivers’ ability to differentiate between truthful and deceptive messages has indicated that, in most cases, people, including professionals whose tasks involve detection of deceit, perform this task at chance levels (see Vrij, 2008 for a review). Consequently it is not surprising that the idea of using physiological measures for detecting deception has been very appealing to law-enforcement agencies (e.g., Marston, 1917, 1938; Larson, 1932; Reid, 1947; Reid and Inbau, 1977). Indeed, several psychophysiological methods (popularly labeled, “polygraph techniques”) have been developed since the beginning of the twentieth century and the study of psychophysiological detection of deception has attracted a great deal of interest among researchers as well as practitioners and has become an important area of applied psychology (e.g., Reid and Inbau, 1977; Raskin, 1989; Ben-Shakhar and Furedy, 1990; Lykken, 1998; National Research Council, 2003). This interest has considerably increased since the September 11 terror attack in the United States and the subsequent terror activities in Europe (for a review of recent research, see Verschuere et al., 2011; Rosenfeld et al., 2012). Furthermore, the increased need to detect suspects involved in planning and executing terror activities has raised new questions that require new research directions. One of the main goals of this paper is to describe and discuss these new directions.

## Methods of Psychophysiological Detection

The various psychophysiological detection methods that have been developed can be broadly classified into two categories: (1) Methods designed to detect deception, which rely on physiological responses to direct questions (e.g., “did you break into the Jewelry store on Thursday night?”); and (2) methods designed to detect concealed knowledge (e.g., “was the stolen jewel, a golden watch?”, “was it a diamond ring?”). The detection method, most closely associated with the first category, has been labeled the Control (or more recently, comparison) Questions Technique (CQT). The CQT has been the preferable detection method used by law-enforcement agencies in the United States and it has been exported to various other countries. Yet, the CQT has been severely criticized and nowadays it is considered by most researchers as lacking scientific foundation (e.g., Ben-Shakhar, 2002; Iacono and Lykken, 2002; National Research Council, 2003). The major obstacle in any attempt to detect deception directly is that there is no specific and unique response associated with deception and under realistic police investigations both deceptive and honest suspects are highly aroused by the relevant (“Did you do it?”) questions and thus may show similar physiological responses to these questions.

The method designed to detect concealed knowledge was traditionally labeled the guilty knowledge test (GKT, see Lykken, 1959, 1960), but more recently it has been referred to as the concealed information test (CIT, see Verschuere et al., 2011). This test utilizes a series of multiple-choice questions, each having one relevant alternative, also labeled as Probe (e.g., a feature of the crime under investigation) and several neutral (control) alternatives, chosen so that an innocent suspect would not be able to discriminate them from the probe (Lykken, 1998). The relevant alternatives are significant only for knowledgeable (guilty) individuals and there is ample evidence, mostly from psychophysiological research on orienting responses (ORs), indicating that significant stimuli elicit enhanced ORs (e.g., Sokolov, 1963; Gati and Ben-Shakhar, 1990; Siddle, 1991). Thus, if the suspect’s physiological responses to the relevant alternative are consistently larger than to the neutral (or irrelevant) alternatives, knowledge about the event (e.g., crime) is inferred. As long as information about the event has not leaked out to innocent suspects, the probability that an innocent suspect would produce consistently stronger responses to the relevant than to the neutral alternatives depends only on the number of questions and the number of alternative answers per question, and hence it can be controlled such that maximal protection for the innocent is provided. Clearly the detection of concealed information does not necessarily imply that the suspect is deceptive, as other explanations may be offered for the possession of guilty knowledge. Thus, deception or guilt can only be inferred indirectly and they require additional investigation. Although the CIT does rely on solid scientific grounds (e.g., Verschuere and Ben-Shakhar, 2011) it is very rarely used in practice in Western countries and in fact it is routinely used as the standard psychophysiological detection method only in Japan (see Osugi, 2011).

This paper will focus only on the CIT because it is the only psychophysiological method that is properly grounded in scientific research and theory. Both the strength and weaknesses of this technique will be briefly described as well as possible reasons for its limited usage. Finally, I will discuss current and future research directions as well as attempts to increase the usage of the CIT.

### A brief review of CIT research

Concealed information test research can be traced back to the early 1940s and 1950s (e.g., Geldreich, 1941, 1942; Ellson et al., 1952), but two articles published by Lykken (1959, 1960) were the first to make a real impact on the field and enhance interest in the CIT among various research groups. This early research relied on just a single physiological measure, namely skin conductance response (SCR) and demonstrated an impressive ability to detect concealed information. Specifically, Lykken (1959) employed a mock-crime procedure where some subjects committed one or two mock-crimes (the “guilty” subjects) while others (the “innocents”) did not commit any. The results revealed that 88% of the "guilty” subjects were detected while none of the “innocent” subjects were misclassified as “guilty.” Lykken’s (1960) second study relied on a personal items paradigm and used 25 biographical details of 20 subjects, all of whom were correctly detected.

Concealed information test research has expanded in several directions in the following decades. First, the validity of additional autonomic measures, such as changes in respiration and heart rate, was examined (e.g., Thackray and Orne, 1968; Cutrow et al., 1972). For a recent review of CIT studies based on autonomic nervous system (ANS) measures, see Gamer (2011a). Furthermore, in the past two decades, much research interest has been devoted to the use of brain evoked potentials (see Rosenfeld, 2011 for a review) and brain imaging (see Gamer, 2011b; Rosenfeld et al., 2012) for the detection of concealed information. Second, attempts were made to shed light on the theoretical basis of the CIT effect – the enhanced responses elicited by the significant stimuli (e.g., Gustafson and Orne, 1963, 1965; Lieblich et al., 1970; Ben-Shakhar, 1977; Ben-Shakhar and Lieblich, 1982; Verschuere et al., 2004, 2007). Third, many studies examined the effects of various factors on the outcomes of the CIT (e.g., the effect of type of verbal responses to the CIT questions, Kugelmass et al., 1967; Horneman and O’Gorman, 1985; the effect of drugs, Waid et al., 1981a; Iacono et al., 1984). Finally, factors that may limit the applicability of the CIT have been examined (e.g., the vulnerability of the CIT to countermeasures, Ben-Shakhar and Dolev, 1996; Honts et al., 1996; the effect of leakage of critical CIT items to innocent suspects, Bradley and Warfield, 1984; Bradley and Rettinger, 1992).

## The Theoretical Foundation of the CIT

Recently, Verschuere and Ben-Shakhar (2011) reviewed the various theoretical approaches proposed to account for the enhanced autonomic responses to the relevant CIT alternatives. In this paper I will discuss only the main theoretical accounts. As the autonomic measures used in the CIT are components of the OR (see Sokolov, 1963; Lynn, 1966), it is not surprising that this concept has been proposed to account for the CIT effect. Furthermore, Sokolov (1963) and his followers noted that significant stimuli (“signal-value stimuli,” to use Sokolov’s terminology) elicit enhanced ORs with slower habituation and this can account for the enhanced responses to the crime-relevant stimuli observed among knowledgeable (guilty) individuals. The relationship between the CIT effect and OR was highlighted by Lykken (1974) who wrote that, “… for the guilty subject only, the ‘correct’ alternative will have a special significance, an added ‘signal value’ which will tend to produce a stronger orienting reflex than that subject will show to other alternatives (p. 728).”

There is ample evidence supporting the OR account for the CIT effect. First, the physiological response pattern elicited by the relevant CIT items in knowledgeable individuals (e.g., increased SCR, Lykken, 1959; heart-rate deceleration, Verschuere et al., 2004; respiratory suppression, Timm, 1982; and increased pupil dilation, Lubow and Fein, 1996) is typical for the OR. Second, several features characteristic of the OR have been demonstrated, using the CIT paradigm. For example, response habituation has been observed in several CIT studies (e.g., Ben-Shakhar et al., 1975; Balloun and Holmes, 1979; Verschuere et al., 2005). In addition, as predicted by OR theory, the CIT effect has been demonstrated to increase when the critical items are less frequently presented (e.g., Ben-Shakhar, 1977). Forth, the information processing view of orienting states that the OR serves to allow more elaborate processing of the OR-eliciting stimulus (Kahneman, 1973; Wagner, 1978; Öhman, 1992). Research demonstrating positive correlations between OR and later recall of the stimulus material supports this view (e.g., Corteen, 1969). Indeed, several CIT studies found a positive association between recall and detection efficiency (e.g., Waid et al., 1978, 1981b; Iacono et al., 1984; Carmel et al., 2003; Verschuere et al., 2007).

On the other hand, some research findings are hard to reconcile with the OR theory. For example, heart-rate deceleration elicited by relevant CIT items may last for 15 s, whereas according to OR theory heart rate typically decelerates 1–5 s after the onset of the OR-eliciting stimulus, and then returns to baseline (Richards and Casey, 1992). In addition, although OR theory predicts greater startle modulation to the relevant than to the irrelevant items, Verschuere et al. (2007) failed to support this prediction and proposed an alternative hypothesis, namely response inhibition, to explain the startle data. Processes other than orienting may contribute to physiological responding in the CIT, and response inhibition seems a reasonable candidate. This account is also supported by recent fMRI research (see Gamer et al., 2007).

## The Validity of the CIT

Although the initial studies reported by Lykken (1959, 1960) produced impressive validity estimates for the CIT based on SCR, the results of subsequent studies that used both SCR and additional ANS measures were less uniform. The best method for evaluating research results across many studies is meta analysis (e.g., Hunter and Schmidt, 1990). Indeed two meta analytic studies published last decade (MacLaren, 2001; Ben-Shakhar and Elaad, 2003) demonstrated a relatively large mean effect size (Cohen’s *d*) for the CIT based on SCRs. For example, Ben-Shakhar and Elaad (2003) covered 80 laboratory studies, which included 169 experimental conditions with a total of 5198 participants tested under a variety of CIT paradigms (e.g., card test, mock-crime) and reported an overall average effect size of 1.55. They further showed that studies relying on the mock-crime paradigm, which seems more relevant for field applications than other paradigms, produced an average effect size of 2.1.

However both meta analyses relied only on a single measure and as more studies using additional measures were published during the last decade, it is more informative to describe a more recent meta analysis that included four measures (Meijer et al., 2012). In addition to SCR, this meta analysis included studies that measured respiration line length (RLL, see e.g., Timm, 1987), heart-rate deceleration (e.g., Ambach et al., 2011), and the P300 component of the event-related potential (e.g., Rosenfeld et al., 1988; Farwell and Donchin, 1991). Meijer et al. (2012) included in their meta analysis two CIT paradigms (the mock-crime and the personal items paradigms) and several measures of detection efficiency. In addition to the average Cohen’s *d* they computed the variance of *d* across studies and subtracted from it the variance that would be expected from sampling errors. The residual variance represents true differences among the studies.

The main results of this meta analysis indicated that the four measures differ significantly in their detection efficiency. Specifically, the P300 measure outperformed all three ANS measures, with an average *d* of 2.55, but it should be noted that 80% of the P00 studies, included in this meta analysis, came from a single laboratory (of J. P. Rosenfeld) that has been most active in the past two decades. The HR measure was the least effective of all four measures that have been examined (with an average d of 0.88), but it is important to note that even this *d* value is considered a large effect size (see Cohen, 1988). Moreover, several studies demonstrated that a combination of several ANS measures outperforms the best single measure (e.g., Ben-Shakhar and Dolev, 1996; Ben-Shakhar and Elaad, 2002; Gamer et al., 2008) and from this respect the *d* value of 1.73 obtained for the SCR measure can be considered as an underestimate of detection efficiency with ANS measures. The results of this meta analysis also revealed a considerable residual variance for the SCR and the P300, which means that real differences between studies exist for these measures.

Indeed, several moderating factors were identified for the SCR measure. Specifically, two factors that were also identified by Ben-Shakhar and Elaad (2003), namely motivation to avoid detection and the number of CIT questions mediate the SCR effect size. In experimental conditions that employed either incentive or instructions to avoid detection, the average *d* was 1.89 as compared with an average of 1.45 observed under law motivation conditions. In addition, when the number of CIT questions used was at least six the average *d* was 1.99 as compared with 1.45 when a smaller number of questions were used. These two moderators may be very important for the application of the CIT because real-life investigations are clearly associated with very high levels of motivation to avoid detection and because investigators can increase detection efficiency by making efforts to identify as many appropriate critical items as possible. The number of ERP studies (32) was too small to allow for an analysis of moderators. In addition, motivation was not manipulated in ERP studies and the number of questions used was more or less uniform.

However, although this meta analysis, as well the previous meta analytic studies (MacLaren, 2001; Ben-Shakhar and Elaad, 2003), demonstrated very large effect sizes, it should be emphasized that only laboratory experiments were analyzed and it is questionable whether the results of CIT experiments can be generalized to realistic criminal investigations. Unfortunately, only two field CIT studies were reported in the scientific literature (Elaad, 1990; Elaad et al., 1992). The results of these studies, which were based on criminal cases investigated by the Israeli Police, showed that while the rates of false-positive errors were as low as those reported in laboratory experiments (2% in the former study, which relied only on the electrodermal measure, and 5% in the latter study, which utilized a combination of electrodermal and respiration measures), the rates of false-negative errors were much larger (42% in the former study and 20% in the latter). This may imply that CIT experiments have a weak external validity, but it should be noted that the use of the CIT in the criminal cases studied by Elaad (1990) and Elaad et al. (1992) was not optimal. In particular, the mean number of questions used in these field studies (2 and 1.8 in Elaad, 1990 and Elaad et al., 1992, respectively), was much smaller than recommended. In addition, the two field studies were based on CITs that were administered immediately after a CQT, and this might attenuate the sensitivity of the physiological measures due to habituation. Thus, it is possible that the relatively high rates of false-negative errors and lower detection efficiency obtained in these field studies resulted from a non-optimal usage of the CIT.

## Weaknesses and Potential Limitations of the CIT

So far, I have listed several advantages of the CIT over alternative detection methods, namely its solid theoretical foundation, the impressive validity estimates obtained for the CIT in experimental settings and its potential for protecting innocent suspects against false classification. Unfortunately, the CIT has several weaknesses and in this section I will discuss factors that may limit its application. As indicated above, the bulk of CIT studies were conducted in artificial laboratory settings where volunteering participants were requested to commit a mock-crime, with no consequences for their well-being. It is important therefore, to examine the factors that differentiate the experimental setting from real criminal investigations.

### Leakage of critical items

Implementation of the CIT depends on a successful concealment of the critical items. Whereas in mock-crime studies concealment is perfectly guaranteed, in real-life this is not necessarily the case and critical items may leak to innocent suspects, either through the media, or during the course of police interrogations.

Several studies examined the effect of information leakage on the CIT accuracy and particularly on false-positive outcomes. Most of these studies were conducted by Bradley and his colleagues (Bradley and Warfield, 1984; Bradley and Rettinger, 1992; Bradley et al., 1996; see Bradley et al., 2011, for a recent review of the leakage literature). Generally, these studies demonstrated that although informed innocent participants show larger relative responses to the critical items, as compared with uninformed innocents, they could be differentiated from guilty participants. However, two recent studies demonstrated that informed innocents were not differentiated from guilty participants when the CIT was administered immediately after the mock-crime (Gamer et al., 2010; Nahari and Ben-Shakhar, 2011). But when the test was delayed (as is usually the case in realistic criminal investigations), informed innocents showed smaller differential responses to the critical items, as compared with guilty participants. This was mediated in both studies by the fact that informed innocents forgot critical items more than guilty participants.

Several means to reduce the damaging effects of information leakage (in addition to improving police practices) were examined by some researchers. Ben-Shakhar et al. (1999) used target items to which participants had to respond in addition to the critical and the control items. Under this procedure, the rate of false-positive outcomes among informed innocents was somewhat reduced.

Bradley and Warfield (1984) proposed a modified version of the CIT, labeled the guilty action test (GAT), in which the formulation of the questions emphasize actions rather than knowledge (e.g., “Did you kill Mr. X with a gun?, knife?…,” rather than “Was Mr. X killed with a gun?, knife? …”). Under the GAT guilty suspects are deceptive when giving negative answers to these questions, whereas informed innocents are telling the truth. Bradley et al. (1996) directly compared the CIT and the GAT and showed that the GAT significantly reduced the false-positive rates, although these rates were still very high (50%). On the other hand, a more recent study by Gamer (2010) failed to find any differences between the two test formats: In both formats informed innocents were undifferentiated from guilty participants.

Previewing the CIT questions has also been offered as a means to prevent the usage of items that might have leaked. Presenting the CIT questions prior to the test may provide examinees with an opportunity to explain that they are familiar with certain items (e.g., they were mentioned in prior interrogations). Verschuere and Crombez (2008) demonstrated that previewing CIT items does not reduce the test’s validity. Clearly, leakage of critical information is a major threat to the validity of the CIT and the test should not be used when critical items were leaked. No information is available about the extent to which critical items are being leaked in police investigations, but the results of the two field studies reported by Elaad and his colleagues (Elaad, 1990; Elaad et al., 1992) were encouraging with this respect, as in both studies the false-positive rates were small, indicating that at least in these criminal cases critical information did not leak.

### The effects of countermeasures

While leakage of critical information may affect false-positive rates, other factors that can increase false-negatives were also identified in previous research. Specifically, several studies demonstrated that the CIT is vulnerable to countermeasures, namely deliberate techniques that might be used by suspects to alter their physiological reactions in order to avoid detection. Several countermeasure techniques have been experimentally examined (e.g., Kubis, 1962; Elaad and Ben-Shakhar, 1991; Ben-Shakhar and Dolev, 1996; Honts et al., 1996; see a recent review of the countermeasure literature in Ben-Shakhar, 2011), but countermeasures were most effective when subjects attempted to create or enhanced responses to the neutral items. This can be achieved either by physical (subjects can bite their tongue to inflict pain when the control items are presented) or by mental means (recalling exciting and emotional memories, or exercising mental activities during presentation of control items). Mental countermeasures may be most harmful because they cannot be detected by the examiners. Two studies examined the effects of mental countermeasures on the outcomes of the CIT (Ben-Shakhar and Dolev, 1996; Honts et al., 1996) and demonstrated a significant reduction in SCR detection efficiency when these countermeasures were applied. However, no countermeasure effects were observed in these studies when the RLL was used as the detection measure.

Clearly, both physical and mental countermeasures require some sophistication and certain knowledge. However, there is an extensive literature in which ANS-based polygraph procedures including effective countermeasure techniques are described in great detail. Thus, the danger that interested individuals may gain the necessary understanding in order to use countermeasures is a real one.

Several researches reported that even CIT based on the P300 component of the event-related potential may be vulnerable to countermeasures (e.g., Rosenfeld et al., 2004; Mertens and Allen, 2008). To overcome this difficulty, Rosenfeld et al. (2008) developed a novel P300 protocol called the *Complex Trial Protocol* which temporally separates the presentation of probe or irrelevant from target or non-target. Several studies reported by Rosenfeld and his colleagues demonstrated that this protocol was indeed highly resistant against both mental and physical countermeasures (Rosenfeld et al., 2008; Meixner and Rosenfeld, 2010; Rosenfeld and Labkovsky, 2010; Winograd and Rosenfeld, 2011). Clearly these studies should be replicated in other laboratories, but they indicate that CIT based ERPs may be immune against countermeasures and as ERPs are associated with very large effect size (see Meijer et al., 2012) they may have an excellent potential as an applied detection method.

### The role of perception and memory of crime-related items on CIT validity

A successful implementation of the CIT in the criminal investigation context depends on the identification of a sufficient number of salient features of the crime, features that are likely to be noticed by the perpetrator and stored in memory. Unfortunately, the bulk of CIT research has been conducted in artificial settings where it was guaranteed that participants memorized all critical features of a mock-crime. Furthermore, the CIT is typically administered immediately after participants committed the mock-crime, whereas in realistic criminal investigations polygraph tests are administered after a relatively long delay. Thus, the external and ecological validity of mock-crime studies seem highly questionable. Recently, three studies examined the role of memory for critical items on the CIT’s outcomes (Carmel et al., 2003; Gamer et al., 2010; Nahari and Ben-Shakhar, 2011). These studies revealed that when the CIT is administered one or two weeks after the mock-crime, certain critical items are not recalled and do not elicit differential responses. However, consistent with memory research (e.g., Kensinger, 2007), memory loss occurs mostly with peripheral items (features that are not directly related to the execution of the crime, such as a picture on the wall of the crime scene). Central features, such as type of weapon used, are capable of eliciting large responses even when the test is delayed. Clearly, this line of research that has important practical implications for constructing proper CITs should be continued and extended.

### The effects of emotional stress and motivation on CIT validity

Another important difference between the typical experimental setup and realistic criminal investigations is the level of stress experienced by the examinees as well as their motivation to avoid detection. However, there are several indications that these factors are not interfering with the external validity of CIT experiments. First, as indicated above, motivation to avoid detection was manipulated in several studies and was generally associated with an increased CIT effect (Ben-Shakhar and Elaad, 2003; Meijer et al., 2012). Thus from this perspective, it seems that the CIT should have even larger detection efficiency in realistic investigations than in laboratory experiments.

Second, two studies (Kugelmass and Lieblich, 1966; Bradley and Janisse, 1981) manipulated the level of stress experienced by subjects while taking the CIT and included levels that seem to resemble realistic situations. Both studies demonstrated that the level of stress had no effect on the outcomes of the CIT. It was concluded that, “within a considerable range of stress no necessary decrease in the detection efficiency of the GSR channel need be expected” (Kugelmass and Lieblich, 1966, p. 215). Thus, on the basis of these two studies it seems that detection efficiency estimated in laboratory experiments can be generalized to situations characterized by much higher levels of motivation and stress.

Third, recently Peth et al. (2012) manipulated the level of stress during mock-crime execution and found that level of stress did not affect the relative responses to the critical CIT items with electrodermal, respiration, and cardiovascular measures. Furthermore, the data revealed that under the high arousal level, detection efficiency based on central items tended to be unaffected by delaying the test. The authors concluded that, “emotional arousal might facilitate the detection of concealed information sometime after the crime occurred” (Peth et al., 2012, p. 381).

## Current Usage of the CIT in Practice

As mentioned above, despite its many advantages, the CIT is hardly used in criminal investigations in the West, whereas the much more controversial, CQT is used extensively in the United States and several other countries. The limitations of the CIT, listed in the previous section have been offered as an explanation for this state of affairs. Krapohl (2011) discussed various factors that limit the applicability of the CIT and classified them into two categories, practical and cultural limitations. The practical factors relate to the difficulty in identifying a sufficient number of salient features of a crime and protecting them from leaking as well as the vulnerability of the CIT to countermeasures (although the CQT is as vulnerable to countermeasures as the CIT, e.g., Honts et al., 1994). Podlesny (1993) made similar arguments and estimated that the CIT might have been used in only 13.1% of FBI cases for which polygraphs have been used. This estimate is based on the assumption that at least four different CIT questions are required to construct a CIT.

However, it is difficult to reconcile these arguments and estimates with the fact that the CIT has been used for many decades by the Japanese police as the standard polygraph method. Approximately 5000 CITs are administered annually in Japan and this method has even been used as admissible evidence in the Japanese criminal courts (Hira and Furumitsu, 2002; Nakayama, 2002; Osugi, 2011). Therefore, it seems more reasonable that the cultural factors may provide a better explanation for these differences in the application of the CIT. Indeed, Krapohl (2011) suggested that even if the practical difficulties were resolved, “the expanded use of the CIT would still face resistance from some experienced polygraph examiners who, wedded to the methods they learned in polygraph school, find such a radical departure from the CQT protocol unsettling and unnecessary” (Krapohl, 2011, p. 160). He added that only 5 out of the 20 certified polygraph schools in the U.S. formally teach the CIT.

There is a huge gap between scientists and practitioners in this area and while the bulk of the scientific community regard the CQT as a non-scientific method, most practitioners believe it is highly accurate. A possible explanation for this gap was offered by Ben-Shakhar (1991) who argued that the belief of practitioners in the validity of the CQT reflects a biased decision process. Specifically, polygraph examiners are affected by the confirmation bias (e.g., Nisbett and Ross, 1980; Darley and Gross, 1983) when they administer the CQT and evaluate the physiological responses. As a result, the outcomes of the CQT are typically consistent with the examiners’ *a priori* hypotheses and this creates a strong illusion of validity (see Einhorn and Hogarth, 1978). In addition, the CQT is often used to extract confessions (Furedy and Liss, 1986), and naturally investigators make efforts to extract confessions only when they believe that the suspect is guilty. Thus, confessions made after a CQT are typically associated with an incriminating CQT’s outcome (Iacono, 1991) and this is another factor that contributes to the illusion of validity. Finally, Western practitioners may have been influenced by the positive results of controlled mock-crime experiments that generally supported the CQT’s validity (although their weak external validity does not allow for generalizing their results, see Ben-Shakhar, 2002).

In addition to the strong belief of polygraph examiners in the CQT’s validity, it should be noted that it is much easier to formulate CQT questions than to identify salient features of a crime and as the CQT is a test of deception, it can be used in all types of criminal cases. Thus, practitioners in most countries do not feel that the CQT need to be replaced.

## Future Directions in Research and Practice

### The need for field-validity studies

In the previous section I discussed several factors differentiating the artificial experimental setting from that of realistic criminal investigations. Clearly, the best approach would be to examine the validity of the CIT as practiced with real suspects. However, as indicated above only two field-validity studies were published so far (Elaad, 1990; Elaad et al., 1992). This unfortunate situation may be explained by the difficulties involved in conducting proper field studies in this area. Specifically, a ground truth criterion is typically unavailable and the use of confessions is problematic because they may depend on the test’s outcomes (see Iacono, 1991). Nevertheless, efforts must be made to overcome these difficulties and the natural setting for such studies seems to be Japanese criminal investigations arena because the CIT is the standard polygraph method used in Japan and because Japanese polygraph investigators have the proper scientific training (Osugi, 2011). The application of the CIT by Japanese Police meets very high standards. Specifically, it typically rests on five different questions (as opposed to an average of about two in the Israeli Police studies), each repeated five times and on four physiological measures (as opposed to one or two in the Israeli studies). Furthermore, from the description of how the CIT is conducted by the Japanese Police (Osugi, 2011), it seems that CITs are conducted independently of other criminal investigations and it is not used as a means to elicit confessions. Such studies would shed light on the validity of the CIT in practice.

### Examining additional physiological and behavioral measures

#### The use of brain imaging in the CIT

The validity of additional measures that can be incorporated into the CIT may also be important. A great research interest has recently been directed to the use of brain imaging for the detection of deception. These studies used a variety of research paradigms and were focused primarily on the search of brain regions that are differentially activated when subjects give deceptive versus truthful responses. For example, the differentiation of deception (DOD) paradigm, designed to isolate deception and examine processes associated with deception (e.g., Furedy et al., 1988), was often used in fMRI research. Other studies used variations of the CIT paradigm (primarily, the card test and the personal item paradigm), to examine brain activation when critical information is concealed. The results based on group data were not uniform and even studies using similar experimental procedures failed to fully replicate their findings, but most studies found regions in the prefrontal cortex being more activated when deceiving or concealing knowledge (see recent reviews by Gamer, 2011b; Rosenfeld et al., 2012). These studies are important from a theoretical perspective as they may shed light on brain mechanisms associated with deception, but from a practical perspective it is important to examine the efficiency of fMRI in classifying individuals as concealing critical information. Only very few studies assessed the validity of the CIT with fMRI. The results of these studies, as summarized by Rosenfeld et al. (2012), indicate that the average sensitivity and specificity were, about 86 and 92%, respectively. These figures are more or less similar to those obtained with ERPs (Meijer et al., 2012) and also to those obtained with a combination of ANS measures (see Gamer et al., 2008). Thus, given the complexity of fMRI measurement relative to ANS and ERP measures, it is highly questionable whether fMRI would have a practical utility as a field detection method. In addition, detection of concealed information with fMRI is vulnerable to all the threats mentioned earlier and the generalizability of the few published studies is questionable. For example, Ganis et al. (2011) demonstrated that when subjects applied countermeasures CIT detection accuracy with fMRI dropped from 100 to only 33%.

#### The use of behavioral measures

Several behavioral measures can be used for detecting concealed information with the CIT, but these measures have received relatively little research attention and definitely should be more thoroughly explored. Examining response latency (or response time-RT) to critical and neutral items is a natural candidate for providing useful information that can distinguish between knowledgeable and unknowledgeable (innocent) individuals because significant stimuli capture attention and thus require more processing time. Indeed, RT has been included in many ERP studies using the oddball paradigm (e.g., Farwell and Donchin, 1991) and showed the expected effect (enhanced RTs to critical items among knowledgeable participants). Moreover, Allen et al. (1992) reported a slightly better performance of the behavioral measures (response time and number of errors) as compared with the ERP measures. Seymour et al. (2000) were the first to examine RTs as a sole index for information concealment and concluded that RTs can serve as a simple alternative to the physiological measures typically used in the CIT. However, the question of weather RTs have incremental validity over ANS or ERP measures has not been resolved yet and studies using different paradigms produced different results (e.g., Gronau et al., 2005; Verschuere et al., 2009). In their review of the research on the use of RTs in the CIT, Verschuere and De Houwer (2011) argued that paradigms based on a manipulation of relevant stimulus-response compatibility, such as the oddball task are effective, whereas tasks that do not manipulate relevant stimulus-response compatibility, such as the modified Stroop used by Gronau et al. (2005) have not produced robust response latency differences between concealed and control items. Clearly, this is an important hypothesis that deserves further research. Similarly, the vulnerability of RT to countermeasure manipulations should be thoroughly examined.

#### The symptom validity test

This test may be promising because it is based on an entirely different rationale than that underlying both physiological and RT measures. Specifically, the SVT is based on asking examinees, who deny knowledge of the critical items, to guess these items. Effective concealment is possible when guessing is random (i.e., where the critical alternative is guessed with the same probability as all other alternatives), but producing random guesses may be very difficult for those who are actually aware of the true alternatives. Consequently the outcome of multiple guessing attempts may differentiate knowledgeable (who would not be able to produce random guessing) and unknowledgeable examinees (whose guesses will be random). The SVT has been used to detect malingering in various contexts (e.g., Merckelbach et al., 2002; Verschuere et al., 2008) and recently it has been adopted for the CIT (Meijer et al., 2007; Nahari and Ben-Shakhar, 2011). These studies demonstrated that the SVT can improve detection efficiency when combined with ANS measures. Once again, much more research is required to determine the practical utility of the SVT.

### The potential use of the CIT in the anti-terror campaign

The increased terror activities during the last decade have raised an increased interest in detection methods in general, and particularly in the CIT. The use of the CIT to detect individuals and groups involved in terror activities has raised new questions. First, suspects in terror activities are often being interrogated about their plans, rather than about crimes already committed. Thus, one question that deserves careful research is whether detecting past *actions* is equivalent to detecting future *intentions*. Two initial studies have already examined this question. Meijer et al. (2010a) conducted a systematic comparison between committing a mock-crime and planning a mock-crime. These authors demonstrated that the CIT with the SCR measure was similarly effective in both conditions, suggesting that the CIT can be used to detect mal-intentions. This conclusion is also supported by recent findings reported by Meixner and Rosenfeld (2011) showing impressive detection efficiency of the P300-based-CIT with participants who planned a mock terrorist attack. Clearly, this line of research should be continued and elaborated.

A second, related question is whether the CIT can be applied to cases where the precise details are *not* available to the investigators. For example, the Japanese Police applies the CIT in some cases to retrieve information that is unavailable to the investigators (e.g., finding the location of a murder weapon). This application of the CIT, termed “the Searching CIT” (SCIT), is described in detail by Osugi (2011). The SCIT may be applied in the anti-terror campaign. For example, imagine a terrorist group planting a bomb in a certain location unknown to the investigators. Can this location be detected when suspects are identified and tested using the SCIT, to prevent an upcoming explosion? Clearly the use of the SCIT requires some *a priori* knowledge (e.g., possible terror targets) and therefore can be applied only when some intelligence information is available to the investigative authorities. Although the SCIT is being used by the Japanese Police, research examining the validity of this method, as used in Japan is unavailable.

However, initial research on the SCIT has recently emerged. Meijer et al. (2010b) examined the SCIT with the electrodermal measure. They tested 12 participants, who were informed about the details of a planned terror attack, where these details were not known to the investigator (though it was assumed that the terror-related details are among the different alternatives included in the test). Relying upon group averages, these researchers were able to identify the correct alternative in each of the three SCIT questions used. However, this study is of limited external validity because all participants were exposed to the critical items, whereas in most real-life cases, some suspects may be innocent (unaware of the critical items). For example, in the terror attack example, some suspects may be only partially aware of the critical information, or they may be innocent altogether (not belonging to the terror organization). Therefore, it is important to test the SCIT validity under conditions in which suspects’ status (i.e., knowledgeable or unknowledgeable) is unknown to the investigator. Meixner and Rosenfeld (2011) were the first who examined the SCIT with both “guilty” and “innocent” participants. This study used the P300 component of the event-related brain potentials and compared the largest average P300 amplitude of each participant with the second largest response. Detection was made at the individual participant level and 10 out of the 12 knowledgeable participants were correctly detected with no false positives. This yielded an area under the receiver operating characteristic (ROC) curve of 0.979. Additionally, 58% (21 out of 36) critical items were correctly detected.

A different approach was recently adopted by Breska et al. (2012) who examined several algorithms designed to detect the critical items as well as differentiate between knowledgeable and unknowledgeable participants in the SCIT. They reanalyzed three data sets from previous, published CIT studies, assuming that the critical items are unknown to the investigators, but are included among the alternative items presented to the subjects. Specifically, they examined two classes of algorithms. The first class was based on averaging responses across subjects to identify critical items and then on averaging responses across the identified critical items to identify knowledgeable subjects. The second class was based on the correlations between the response profiles of all subject-pairs and applied a principle component analysis to decompose the correlation matrix into its principal components. The detection score was defined as the coefficient of each subject on the component explaining the largest portion of the variance. The results revealed that in most cases all critical items were correctly identified and the efficiency of differentiation between knowledgeable and unknowledgeable subjects in the SCIT (indexed by the area under the ROC curve) approached that of the standard CIT, for both classes of algorithms. In addition, the robustness of these results to variations in the number of knowledgeable and unknowledgeable subjects in the sample was examined. This analysis demonstrated that the performance of these algorithms is relatively robust to changes in the number of individuals examined in each group, provided that at least two (but desirably five or more) knowledgeable examinees are included. Although these results seem promising, the validity of the SCIT should be examined in new experiments involving groups planning illegal activities.

## Conclusion and Recommendations

This paper focused on the CIT and discussed its strength and weaknesses as well as several new potential applications of this method and future research directions. The limited application of the CIT was explained by several practical factors related to its weaknesses and by cultural factors. As the CIT seems to be the only scientifically based detection method, with impressive validity estimates observed in controlled, laboratory studies, it is important to suggest ways to overcome its difficulties and expand its usage. Thus, in this final section I will list several recommendations that may enhance the applicability of the CIT.

*Identifying a sufficient number of salient crime-features*: Recent research suggested that the CIT performs best with central features of the crime, especially when the test is delayed (Carmel et al., 2003; Gamer et al., 2010; Nahari and Ben-Shakhar, 2011). This poses a great challenge because it has been suggested that at least five different CIT questions should be formulated (Lykken, 1988; Ben-Shakhar and Elaad, 2003). Two approaches may be offered to overcome this difficulty. First, although multiple questions are definitely desired, two studies demonstrated that the CIT can be successfully used with much fewer questions, and even with a single question, provided that questions are repeated several times and that a combination of several physiological measures is used (Elaad and Ben-Shakhar, 1997; Ben-Shakhar and Elaad, 2002). Second, the criminal investigation process should be modified, such that polygraph examiners would be able to inspect the crime scene soon after a crime was committed, as practiced by the Japanese National Police.
*Protecting critical items and preventing leakage*: Although the results of the field studies reported by Elaad and his colleagues (Elaad, 1990; Elaad et al., 1992) suggest that leakage of crime-related information did not affect the results of CITs administered by the Israeli police, preventing leakage is essential for a wide application of the CIT. Some research results described earlier (Bradley et al., 1996; Ben-Shakhar et al., 1999) offered methods to reduce the effects of information leakage. However, even with these methods false-positive outcomes among knowledgeable innocent subjects were too high to tolerate. Thus, it seems that the only solution to this problem is to modify police practices, such that critical features of the event are identified and concealed at the outset of the investigation, as a standard investigative practice and that the CIT questions will be previewed by the suspects.
3. *Dealing with countermeasures*: A possible approach for dealing with countermeasure manipulations is the use of the CIT with event-related potentials, rather than autonomic measures. Although initial studies suggested that ERPs are vulnerable to countermeasures (Mertens and Allen, 2008; Rosenfeld et al., 2004), more recent studies using the complex trial protocol showed impressive detection efficiency both when participants applied physical and mental countermeasures and under a non-countermeasure condition (Rosenfeld et al., 2008; Meixner and Rosenfeld, 2010; Rosenfeld and Labkovsky, 2010). In addition, it is important to note that detection efficiency with ERP measures have been demonstrated to be significantly better than that obtained with ANS measures (Meijer et al., 2012). A different approach for dealing with countermeasures was adopted by Elaad and his colleagues who examined several covert respiration measures, with the idea that examinees who are unaware of the fact that they are connected to a polygraph will not be motivated to apply countermeasures (e.g., Elaad and Ben-Shakhar, 2008). However, this idea raises ethical questions that may severely limit or even prohibit its use (for a review of research on covert measures, see Elaad, 2011). More recently, two studies examined whether the CIT can be applied when the questions are presented subliminally and masked (Lui and Rosenfeld, 2009; Maoz et al., 2012). The rationale is similar to the use of covert measures, but it is unclear whether the potential advantage of using invisible stimuli in combating countermeasures, outweighs the cost of reducing detection efficiency as observed by Maoz et al. (2012) under subliminal presentation conditions.
*Future research directions*: Clearly, all the above recommendations require additional research. For example, the complex trial protocol should be further examined in various laboratories. Similarly, the idea that memory of central crime details is stable over time and unaffected by emotional stress needs further research. Finally, it is essential to examine these factors under realistic conditions, with real criminal suspects.

## Conflict of Interest Statement

The author declares that the research was conducted in the absence of any commercial or financial relationships that could be construed as a potential conflict of interest.
